# *N*′-(3-Sulfanyl­idene-3,4-di­hydro­quinoxalin-2-yl)benzohydrazide di­methyl­formamide monosolvate

**DOI:** 10.1107/S1600536813019181

**Published:** 2013-07-17

**Authors:** Asmae Zanzoul, El Mokhtar Essassi, Geneviève Pratviel, Mohamed Saadi, Lahcen El Ammari

**Affiliations:** a Laboratoire de Chimie Organique Hétérocyclique, URAC 21, Pôle de Compétences Pharmacochimie, Université Mohammed V-Agdal, BP 1014 Avenue, Ibn Batouta, Rabat, Morocco; b Laboratoire de Chimie de Coordination du CNRS 205, Route de Narbonne 31077, Toulouse, France; c Laboratoire de Chimie du Solide Appliquée, Faculté des Sciences, Université Mohammed V-Agdal, Avenue Ibn Battouta, BP 1014, Rabat, Morocco

## Abstract

The 2-sulfanyl­idene-3,4-di­hydro­quinoxalin-2-yl ring system of the title solvate, C_15_H_12_N_4_OS·C_3_H_7_NO, is essentially planar, the maximum deviation from the mean plane being 0.024 (2) Å for the thione C atom. The mean plane through the fused-ring system is almost perpendicular to the terminal phenyl ring, as indicated by the dihedral angle of 70.05 (8)°. In the crystal, the main and solvent mol­ecules are linked by N—H⋯O hydrogen bonds, forming a layer parallel to (010).

## Related literature

For potential applications of quinoxaline derivatives, see: Cheon *et al.* (2004 ▶); Jackson *et al.* (1991 ▶); Benzeid *et al.* (2012 ▶).
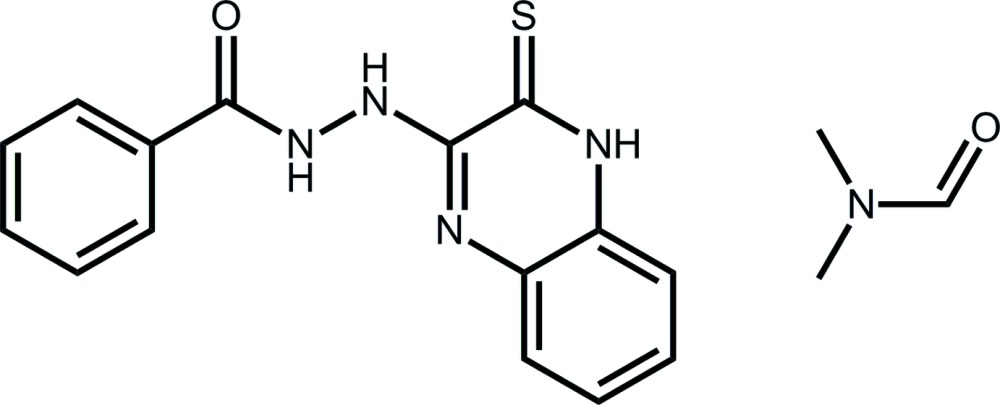


## Experimental

### 

#### Crystal data

C_15_H_12_N_4_OS·C_3_H_7_NO
*M* *_r_* = 369.44
Monoclinic, 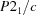

*a* = 10.4053 (2) Å
*b* = 16.8563 (5) Å
*c* = 10.3624 (2) Å
β = 100.882 (2)°
*V* = 1784.83 (7) Å^3^
*Z* = 4
Mo *K*α radiation
μ = 0.21 mm^−1^
*T* = 180 K
0.20 × 0.12 × 0.04 mm

#### Data collection

Oxford Diffraction Xcalibur (Eos, Gemini ultra) diffractometer
Absorption correction: multi-scan (*CrysAlis RED*; Oxford Diffraction, 2012 ▶) *T*_min_ = 0.960, *T*_max_ = 0.992
15848 measured reflections
4153 independent reflections
3090 reflections with *I* > 2σ(*I*)
*R*_int_ = 0.043

#### Refinement

*R*[*F* ^2^ > 2σ(*F* ^2^)] = 0.047
*wR*(*F* ^2^) = 0.119
*S* = 1.03
4153 reflections
235 parameters
H-atom parameters constrained
Δρ_max_ = 0.30 e Å^−3^
Δρ_min_ = −0.25 e Å^−3^

### 

Data collection: *CrysAlis CCD* (Oxford Diffraction, 2012 ▶); cell refinement: *CrysAlis RED* (Oxford Diffraction, 2012 ▶); data reduction: *CrysAlis RED*; program(s) used to solve structure: *SIR97* (Altomare *et al.*, 1999 ▶); program(s) used to refine structure: *SHELXL97* (Sheldrick, 2008 ▶); molecular graphics: *ORTEP-3 for Windows* (Farrugia, 2012 ▶); software used to prepare material for publication: *WinGX* (Farrugia, 2012 ▶) and *publCIF* (Westrip, 2010 ▶).

## Supplementary Material

Crystal structure: contains datablock(s) I, global. DOI: 10.1107/S1600536813019181/tk5236sup1.cif

Structure factors: contains datablock(s) I. DOI: 10.1107/S1600536813019181/tk5236Isup2.hkl

Supplementary material file. DOI: 10.1107/S1600536813019181/tk5236Isup3.cml

Additional supplementary materials:  crystallographic information; 3D view; checkCIF report

## Figures and Tables

**Table 1:** Hydrogen-bond geometry (Å, °)

*D*—H⋯*A*	*D*—H	H⋯*A*	*D*⋯*A*	*D*—H⋯*A*
N3—H3*N*⋯O2^i^	0.88	2.14	2.906 (2)	146
N4—H4*N*⋯O2^ii^	0.88	2.04	2.906 (2)	166
N1—H1⋯O1^iii^	0.88	2.01	2.8331 (19)	154

Symmetry codes: (i) 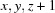; (ii) 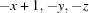; (iii) 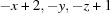.

**Fig. 1.**
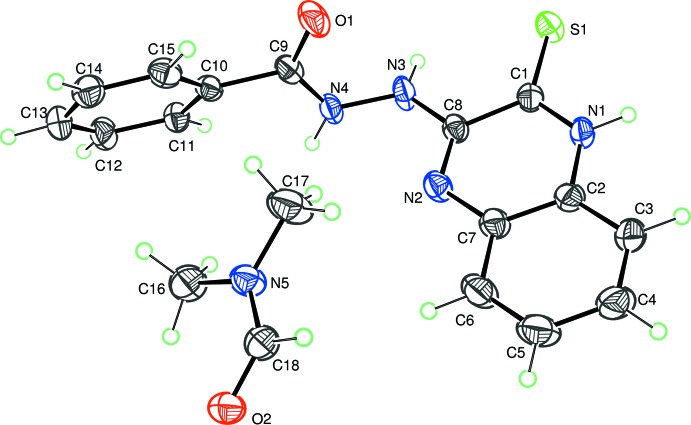
Molecular structures of the components of the title compound showing the atom-labelling scheme. Displacement ellipsoids are drawn at the 50% probability level.

## supplementary crystallographic information

### Comment

Quinoxaline derivatives have been discovered as leads for a novel series of dipeptidyl peptidase-IV molecules (Cheon *et al.*, 2004). They are also used as ligands for the strychnine-insensitive glycine site (Jackson *et al.*, 1991) and as new fluorescent probes for amyloid-β fibrils (Benzeid *et al.*, 2012).

The crystal structure of title compound is build up from two fused six-membered rings (N1, N2 C1–C8) linked to a benzohydrazide system (N3, N4, O1, C9–C15) and a dimethylformamide solvent molecule as shown in Fig. 1. The fused rings system is almost planar with the maximum deviation from the mean plane being -0.024 (2) Å for the C1 atom. The dihedral angle between the terminal phenyl ring and the fused ring system is 70.05 (8)°. In the crystal structure, the molecules and the solvent are linked by N—H···O hydrogen bond to form a layer parallel to (0 1 0), Table 1.

### Experimental

A mixture of quinoxaline-2,3-dithione (1 g, 5.15 10 ^-3^ mol), benzhydrazide (1.4 g, 0.01 mol) and DMF (40 ml) was boiled under reflux for 48 h. The volume of DMF was reduced under reduced pressure (2 ml) and the residue was taken up into 100 ml of diethyl ether. An oily product precipitated quickly after addition of diethyl ether. After centrifugation the diethyl ether phase was recovered and the precipitate (oily product) was washed with diethyl ether (2 x 3 ml). The product crystallized in the diethyl ether phase overnight at room temperature. Crystals were collected, washed with diethyl ether (5 ml) and dried under vacuum. Yield: 700 mg, 46%.

### Refinement

All H atoms could be located in a difference Fourier map. However, they were placed in calculated positions with C—H = 0.95 Å (aromatic), N—H = 0.88 Å and C—H = 0.98 Å (methyl) and refined as riding on their parent atoms with *U*_iso_(H) = 1.2 *U*_eq_(aromatic and N) and *U*_iso_(H) = 1.5 *U*_eq_(methyl).

### Crystal data

**Table:**

C_15_H_12_N_4_OS·C_3_H_7_NO	*F*(000) = 776
*M**_r_* = 369.44	*D*_x_ = 1.375 Mg m^−^^3^
Monoclinic, *P*2_1_/*c*	Mo *K*α radiation, λ = 0.71073 Å
Hall symbol: -P 2ybc	Cell parameters from 4153 reflections
*a* = 10.4053 (2) Å	θ = 3.1–27.9°
*b* = 16.8563 (5) Å	µ = 0.21 mm^−^^1^
*c* = 10.3624 (2) Å	*T* = 180 K
β = 100.882 (2)°	Plate, yellow
*V* = 1784.83 (7) Å^3^	0.20 × 0.12 × 0.04 mm
*Z* = 4	

### Data collection

**Table:**

Oxford Diffraction Xcalibur (Eos, Gemini ultra) diffractometer	4153 independent reflections
Graphite monochromator	3090 reflections with *I* > 2σ(*I*)
Detector resolution: 16.1978 pixels mm^-1^	*R*_int_ = 0.043
ω scans	θ_max_ = 27.9°, θ_min_ = 3.1°
Absorption correction: multi-scan (*CrysAlis RED*; Oxford Diffraction, 2012)	*h* = −13→13
*T*_min_ = 0.960, *T*_max_ = 0.992	*k* = −21→21
15848 measured reflections	*l* = −13→13

### Refinement

**Table:**

Refinement on *F*^2^	Primary atom site location: structure-invariant direct methods
Least-squares matrix: full	Secondary atom site location: difference Fourier map
*R*[*F*^2^ > 2σ(*F*^2^)] = 0.047	Hydrogen site location: difference Fourier map
*wR*(*F*^2^) = 0.119	H-atom parameters constrained
*S* = 1.03	*w* = 1/[σ^2^(*F*_o_^2^) + (0.0514*P*)^2^ + 0.657*P*] where *P* = (*F*_o_^2^ + 2*F*_c_^2^)/3
4153 reflections	(Δ/σ)_max_ = 0.001
235 parameters	Δρ_max_ = 0.30 e Å^−^^3^
0 restraints	Δρ_min_ = −0.25 e Å^−^^3^

### Special details

**Table:**

Geometry. All s.u.'s (except the s.u. in the dihedral angle between two l.s. planes) are estimated using the full covariance matrix. The cell s.u.'s are taken into account individually in the estimation of s.u.'s in distances, angles and torsion angles; correlations between s.u.'s in cell parameters are only used when they are defined by crystal symmetry. An approximate (isotropic) treatment of cell s.u.'s is used for estimating s.u.'s involving l.s. planes.
Refinement. Refinement of *F*^2^ against ALL reflections. The weighted *R*-factor *wR* and goodness of fit *S* are based on *F*^2^, conventional *R*-factors *R* are based on *F*, with *F* set to zero for negative *F*^2^. The threshold expression of *F*^2^ > 2σ(*F*^2^) is used only for calculating *R*-factors(gt) *etc*. and is not relevant to the choice of reflections for refinement. *R*-factors based on *F*^2^ are statistically about twice as large as those based on *F*, and *R*- factors based on ALL data will be even larger.

### Fractional atomic coordinates and isotropic or equivalent isotropic displacement parameters (Å^2^)

**Table:**

	*x*	*y*	*z*	*U*_iso_*/*U*_eq_	
C1	0.90077 (17)	0.07517 (11)	0.52315 (18)	0.0238 (4)	
C2	0.97172 (18)	0.13196 (12)	0.33400 (19)	0.0277 (4)	
C3	1.0611 (2)	0.18006 (13)	0.2856 (2)	0.0353 (5)	
H3	1.1320	0.2040	0.3438	0.042*	
C4	1.0448 (2)	0.19226 (15)	0.1524 (2)	0.0451 (6)	
H4	1.1046	0.2249	0.1179	0.054*	
C5	0.9401 (2)	0.15666 (16)	0.0673 (2)	0.0475 (6)	
H5	0.9300	0.1649	−0.0248	0.057*	
C6	0.8519 (2)	0.11001 (15)	0.1155 (2)	0.0403 (5)	
H6	0.7808	0.0868	0.0565	0.048*	
C7	0.86557 (18)	0.09631 (12)	0.25101 (19)	0.0289 (4)	
C8	0.79327 (17)	0.03925 (11)	0.42633 (17)	0.0231 (4)	
C9	0.64594 (16)	−0.12910 (12)	0.37671 (17)	0.0245 (4)	
C10	0.53941 (17)	−0.17643 (11)	0.29440 (17)	0.0230 (4)	
C11	0.40700 (17)	−0.15997 (12)	0.28901 (18)	0.0264 (4)	
H11	0.3817	−0.1175	0.3391	0.032*	
C12	0.31276 (19)	−0.20563 (13)	0.2106 (2)	0.0339 (5)	
H12	0.2226	−0.1951	0.2083	0.041*	
C13	0.3494 (2)	−0.26669 (13)	0.1356 (2)	0.0357 (5)	
H13	0.2843	−0.2962	0.0787	0.043*	
C14	0.4802 (2)	−0.28490 (12)	0.14304 (19)	0.0332 (5)	
H14	0.5049	−0.3278	0.0934	0.040*	
C15	0.57487 (19)	−0.24034 (12)	0.22322 (18)	0.0290 (4)	
H15	0.6647	−0.2534	0.2298	0.035*	
C16	0.5145 (2)	−0.09973 (14)	−0.0642 (2)	0.0382 (5)	
H16A	0.4905	−0.0777	0.0155	0.057*	
H16C	0.5032	−0.1575	−0.0649	0.057*	
H16B	0.4582	−0.0768	−0.1418	0.057*	
C17	0.7469 (2)	−0.09952 (17)	0.0508 (2)	0.0474 (6)	
H17A	0.8339	−0.0827	0.0381	0.071*	
H17B	0.7472	−0.1568	0.0666	0.071*	
H17C	0.7246	−0.0716	0.1265	0.071*	
C18	0.6865 (2)	−0.05175 (12)	−0.17353 (19)	0.0314 (4)	
H18	0.7771	−0.0417	−0.1684	0.038*	
N1	0.98526 (15)	0.11775 (9)	0.46798 (15)	0.0263 (4)	
H1	1.0539	0.1380	0.5203	0.032*	
N2	0.77741 (15)	0.04876 (10)	0.29977 (15)	0.0277 (4)	
N3	0.70812 (15)	−0.00590 (10)	0.47938 (15)	0.0306 (4)	
H3N	0.7111	−0.0052	0.5648	0.037*	
N4	0.61647 (15)	−0.05320 (10)	0.39948 (15)	0.0264 (4)	
H4N	0.5395	−0.0336	0.3639	0.032*	
N5	0.65059 (16)	−0.08079 (10)	−0.06642 (15)	0.0312 (4)	
O1	0.75344 (13)	−0.15906 (9)	0.42053 (14)	0.0373 (4)	
O2	0.61339 (14)	−0.03660 (9)	−0.27891 (13)	0.0342 (3)	
S1	0.91904 (5)	0.06336 (3)	0.68523 (5)	0.03039 (15)	

### Atomic displacement parameters (Å^2^)

**Table:**

	*U*^11^	*U*^22^	*U*^33^	*U*^12^	*U*^13^	*U*^23^
C1	0.0196 (8)	0.0200 (9)	0.0310 (10)	0.0026 (7)	0.0027 (7)	−0.0020 (7)
C2	0.0236 (9)	0.0272 (10)	0.0317 (10)	0.0012 (8)	0.0033 (8)	0.0048 (8)
C3	0.0263 (10)	0.0356 (12)	0.0422 (12)	−0.0059 (9)	0.0017 (9)	0.0084 (9)
C4	0.0325 (11)	0.0529 (15)	0.0495 (13)	−0.0053 (10)	0.0066 (10)	0.0223 (11)
C5	0.0351 (12)	0.0684 (18)	0.0373 (12)	−0.0015 (12)	0.0028 (10)	0.0218 (12)
C6	0.0291 (11)	0.0580 (15)	0.0308 (11)	−0.0051 (10)	−0.0018 (9)	0.0078 (10)
C7	0.0219 (9)	0.0338 (11)	0.0306 (10)	−0.0001 (8)	0.0037 (8)	0.0043 (8)
C8	0.0192 (8)	0.0234 (10)	0.0256 (9)	0.0012 (7)	0.0016 (7)	−0.0026 (7)
C9	0.0180 (8)	0.0364 (11)	0.0193 (9)	−0.0036 (8)	0.0043 (7)	0.0022 (8)
C10	0.0222 (8)	0.0265 (10)	0.0197 (8)	−0.0018 (7)	0.0020 (7)	0.0027 (7)
C11	0.0229 (9)	0.0269 (10)	0.0290 (10)	−0.0018 (8)	0.0040 (8)	−0.0027 (8)
C12	0.0233 (9)	0.0340 (12)	0.0411 (11)	−0.0028 (8)	−0.0020 (8)	−0.0018 (9)
C13	0.0369 (11)	0.0318 (12)	0.0340 (11)	−0.0097 (9)	−0.0041 (9)	−0.0025 (9)
C14	0.0454 (12)	0.0270 (11)	0.0274 (10)	0.0004 (9)	0.0072 (9)	−0.0033 (8)
C15	0.0275 (9)	0.0335 (11)	0.0262 (9)	0.0027 (8)	0.0060 (8)	0.0025 (8)
C16	0.0374 (11)	0.0440 (13)	0.0349 (11)	−0.0037 (10)	0.0114 (9)	0.0034 (10)
C17	0.0407 (13)	0.0691 (17)	0.0308 (11)	0.0010 (12)	0.0030 (10)	0.0126 (11)
C18	0.0308 (10)	0.0328 (11)	0.0312 (10)	−0.0021 (9)	0.0075 (8)	0.0017 (8)
N1	0.0198 (7)	0.0260 (9)	0.0312 (9)	−0.0047 (6)	0.0006 (6)	−0.0010 (7)
N2	0.0214 (8)	0.0350 (10)	0.0260 (8)	−0.0024 (7)	0.0025 (6)	−0.0003 (7)
N3	0.0284 (8)	0.0413 (10)	0.0213 (8)	−0.0152 (7)	0.0031 (7)	−0.0067 (7)
N4	0.0199 (7)	0.0328 (9)	0.0246 (8)	−0.0067 (7)	−0.0008 (6)	−0.0023 (7)
N5	0.0300 (9)	0.0392 (10)	0.0241 (8)	0.0000 (7)	0.0048 (7)	0.0041 (7)
O1	0.0192 (7)	0.0457 (9)	0.0432 (8)	0.0007 (6)	−0.0041 (6)	−0.0027 (7)
O2	0.0356 (8)	0.0374 (8)	0.0286 (7)	−0.0017 (6)	0.0033 (6)	0.0069 (6)
S1	0.0267 (2)	0.0378 (3)	0.0253 (3)	−0.0027 (2)	0.00135 (19)	−0.0026 (2)

### Geometric parameters (Å, º)

**Table:**

C1—N1	1.343 (2)	C11—H11	0.9500
C1—C8	1.483 (2)	C12—C13	1.386 (3)
C1—S1	1.6661 (19)	C12—H12	0.9500
C2—N1	1.390 (2)	C13—C14	1.383 (3)
C2—C3	1.396 (3)	C13—H13	0.9500
C2—C7	1.401 (3)	C14—C15	1.384 (3)
C3—C4	1.374 (3)	C14—H14	0.9500
C3—H3	0.9500	C15—H15	0.9500
C4—C5	1.401 (3)	C16—N5	1.456 (3)
C4—H4	0.9500	C16—H16A	0.9800
C5—C6	1.372 (3)	C16—H16C	0.9800
C5—H5	0.9500	C16—H16B	0.9800
C6—C7	1.404 (3)	C17—N5	1.456 (3)
C6—H6	0.9500	C17—H17A	0.9800
C7—N2	1.384 (2)	C17—H17B	0.9800
C8—N2	1.300 (2)	C17—H17C	0.9800
C8—N3	1.360 (2)	C18—O2	1.234 (2)
C9—O1	1.233 (2)	C18—N5	1.330 (3)
C9—N4	1.347 (3)	C18—H18	0.9500
C9—C10	1.495 (2)	N1—H1	0.8800
C10—C15	1.394 (3)	N3—N4	1.390 (2)
C10—C11	1.396 (2)	N3—H3N	0.8800
C11—C12	1.383 (3)	N4—H4N	0.8800
			
N1—C1—C8	113.62 (16)	C14—C13—H13	119.8
N1—C1—S1	122.24 (13)	C12—C13—H13	119.8
C8—C1—S1	124.13 (14)	C13—C14—C15	119.64 (19)
N1—C2—C3	120.68 (17)	C13—C14—H14	120.2
N1—C2—C7	117.32 (17)	C15—C14—H14	120.2
C3—C2—C7	122.00 (18)	C14—C15—C10	120.48 (18)
C4—C3—C2	118.92 (19)	C14—C15—H15	119.8
C4—C3—H3	120.5	C10—C15—H15	119.8
C2—C3—H3	120.5	N5—C16—H16A	109.5
C3—C4—C5	120.2 (2)	N5—C16—H16C	109.5
C3—C4—H4	119.9	H16A—C16—H16C	109.5
C5—C4—H4	119.9	N5—C16—H16B	109.5
C6—C5—C4	120.7 (2)	H16A—C16—H16B	109.5
C6—C5—H5	119.7	H16C—C16—H16B	109.5
C4—C5—H5	119.7	N5—C17—H17A	109.5
C5—C6—C7	120.7 (2)	N5—C17—H17B	109.5
C5—C6—H6	119.7	H17A—C17—H17B	109.5
C7—C6—H6	119.7	N5—C17—H17C	109.5
N2—C7—C2	121.65 (17)	H17A—C17—H17C	109.5
N2—C7—C6	120.77 (17)	H17B—C17—H17C	109.5
C2—C7—C6	117.58 (18)	O2—C18—N5	126.27 (19)
N2—C8—N3	120.54 (16)	O2—C18—H18	116.9
N2—C8—C1	124.59 (17)	N5—C18—H18	116.9
N3—C8—C1	114.87 (16)	C1—N1—C2	124.49 (15)
O1—C9—N4	123.04 (17)	C1—N1—H1	117.8
O1—C9—C10	120.98 (18)	C2—N1—H1	117.8
N4—C9—C10	115.98 (15)	C8—N2—C7	118.23 (16)
C15—C10—C11	119.35 (17)	C8—N3—N4	120.40 (15)
C15—C10—C9	118.19 (16)	C8—N3—H3N	119.8
C11—C10—C9	122.44 (17)	N4—N3—H3N	119.8
C12—C11—C10	119.86 (18)	C9—N4—N3	119.73 (15)
C12—C11—H11	120.1	C9—N4—H4N	120.1
C10—C11—H11	120.1	N3—N4—H4N	120.1
C11—C12—C13	120.21 (19)	C18—N5—C17	121.23 (17)
C11—C12—H12	119.9	C18—N5—C16	121.41 (17)
C13—C12—H12	119.9	C17—N5—C16	117.25 (17)
C14—C13—C12	120.34 (18)		

### Hydrogen-bond geometry (Å, º)

**Table:**

*D*—H···*A*	*D*—H	H···*A*	*D*···*A*	*D*—H···*A*
N3—H3*N*···O2^i^	0.88	2.14	2.906 (2)	146
N4—H4*N*···O2^ii^	0.88	2.04	2.906 (2)	166
N1—H1···O1^iii^	0.88	2.01	2.8331 (19)	154

Symmetry codes: (i) *x*, *y*, *z*+1; (ii) −*x*+1, −*y*, −*z*; (iii) −*x*+2, −*y*, −*z*+1.
